# “We do not go outside, though We want to”: Unequal Access to Public Transport and Transport-Related Social Exclusion of Older Adults in Dhaka, Bangladesh

**DOI:** 10.1177/07334648241231156

**Published:** 2024-02-14

**Authors:** Selim Jahangir, Ajay Bailey, Musleh Uddin Hasan, Shanawez Hossain

**Affiliations:** 1 International Development Studies, Department of Human Geography and Spatial Planning, 8125 Utrecht University, The Netherlands; 2 Transdisciplinary Centre for Qualitative Methods, Prasanna School of Public Health, Manipal Academy of Higher Education, India; 3 Department of Urban and Regional Planning, 61750 Bangladesh University of Engineering & Technology, Dhaka, Bangladesh; 4 Global Studies and Governance, 117116 Independent University, Dhaka, Bangladesh

**Keywords:** accessibility, public transport, social exclusion, older adults, mobility

## Abstract

This study investigated key physical and social barriers to accessing public transport in Dhaka, Bangladesh, and how the unequal accessibility of transport leads to the social exclusion of older adults. Employing a transport disadvantage perspective and drawing on visual surveys and in-depth interviews, the study explores the context and lived experiences of older adults using public transport in their everyday lives. Difficulty in accessing buses due to overcrowding and congestion, struggling to get on rickshaws due to height, avoiding *CNG* (an autorickshaw) and cabs due to high fares, disliking *Laguna* (a small four-wheeler human haulier for carrying passengers) for compact seating arrangements, undesirable behavior, and social attitudes discourage older adults from participating in social activities and produce a feeling of social isolation and exclusion. Hence, more inclusive transport policies are essential in low- and middle-income countries to reduce transport-related social exclusion and improve the well-being of older adults.

**What this paper adds**

• Unequal access to public transport leads to social exclusion.
• Older adults face physical and social barriers to accessing public transportation.
• Age, gender, and low-income influence the accessibility.

**Applications of study findings**

• Sensitizing the transport personnel will encourage older adults to participate in social activities.
• Neighborhood proximity will enable older adults to have better access to public transportation.
• Need to develop an evidence-based, inclusive urban mobility framework for older adults.

## Introduction

Transport disadvantage, in terms of inequalities in accessing public transportation, limits an individual’s mobility to access essential activities (Ma et al., 2018). There is a strong relationship between transport disadvantage and the community’s social exclusion, particularly of low-income groups, older adults, women, and people with disabilities. Transportation-related social exclusion has a variety of implications, including barriers to employment, exclusion from healthcare and other services, and limits to maintaining social relations. Here, transport-related social exclusion refers to the deprivation of older adults from participating in work, availing of health facilities, and engaging in social activities due to inaccessible transport (Rashid & Yigitcanlar, 2015). Accessibility is identified as the ability to access public transportation for essential services in a neighborhood spatial context. This paper explored the accessibility issues facing older adults surrounding the transportation dimension of social exclusion. The present study brought out the neighborhood context of challenges to accessing public transport, on which older adults rely for their mobility. Hence, the study addressed the research questions of what specific challenges older adults encounter while accessing different modes of public transport in their everyday mobility and how various intersectional factors such as age, gender, low income, and neighborhood context influence these challenges.

## Literature Review

Previous studies identified transport-related social exclusion as a critical issue in urban policy challenges (Duvarci et al., 2015). These studies documented that unequal access to transport particularly impacts vulnerable groups, such as people with disabilities and women, and thus leads to their social exclusion. The accessibility barriers depend on individual circumstances and neighborhood spatial contexts (Hine & Mitchell, 2001). The spatial approach focuses on the quality of transport supply for residents’ needs and the inaccessibility of public transport that constrains users from participating in employment, health services, and social interactions (Litman, 2014). Several studies have assessed that lack of access to public transport leads to the social exclusion of older adults, particularly in deprived urban areas (Burns et al., 2011; Tong & Lai, 2016).

A large number of studies on transport-related exclusion investigated how social exclusion is caused by transport supply, where and why it occurs, focusing on specific dimensions such as poverty, age, and physical disability (Currie & Delbosc, 2010). Lucas (2012) explained that transportation disadvantages are caused by high travel costs, time constraints, geographical distance, and transport supply. Similarly, Dempsey et al. (2011) mentioned that inaccessibility is a multi-dimensional factor including physical barriers, frequency of modes, travel time, and attitudes toward participation. Litman (2014) stated that both horizontal and vertical transport equity are essential for a transport-inclusive city, where horizontal equity focuses on transport fairness to access all spaces and vertical equity focuses on intersectionality to access resources.

However, previous studies have focused on the travel time, cost, and policy dimension to see the transport inequity in economically advanced societies (Dempsey et al., 2011), whereas this study focused on the infrastructural and social dimension of transport-related social exclusion of older adults from global south neighborhoods in Dhaka, Bangladesh.

Unequal access to public transport and the subsequent social exclusion of older adults can be explained within the theoretical framework of transport disadvantage developed by Lucas et al. (2016). The model stressed that transport-related social exclusion does not refer only to the inaccessibility of transport services such as availability, frequency, and travel time, but also encompasses social disadvantage caused by low income, age, affordability, social attitude, and participation. The model conceptualized that horizontal transport inequity such as spatial deprivation (hence, neighborhood deprivation) significantly influences an individual’s mobility. However, the transport-related social exclusion model explains that a lack of adequate public transport services drives older adults to use private vehicles further pushing them towards transport disadvantage. Hence, social dimensions including affordability, safety, and behavior towards older adults determine their vertical transport inequity.

Lucas et al. (2016) argued that socioeconomic deprivation and social exclusion are the results of limited mobility and accessibility to public transportation. Many studies in transport behavior literature have documented that older adults with low income are more likely to have lower access to public transportation in Australia (Hurni, 2005), the UK (Lucas, 2012), the United States (Paez et al., 2010), South America (Blanco & Apaolaza, 2018), and South Africa (Lucas, 2011). In addition, Hui and Habib (2017) focused on the accessibility dimension of transport-related social exclusion. They argued that limited mobility restricts individuals’ abilities to access essential goods and basic amenities; and is often linked to high costs, break journeys due to limited available routes, safety issues, long waiting times, prolonged journeys, and inconvenient stops. On the other hand, Priya Uteng (2009) documented the time-use dimension of transport-related social exclusion wherein she stated that working and traveling long hours reduce people’s social participation.

The literature on transport and health studies maintained that transport-related social exclusion adversely affects the mental health of older adults (Dharmowijoyo et al., 2020). In addition, Rachele et al. (2017) argued that older adults living in disadvantaged neighborhoods reported poorer health outcomes. Similarly, Ma et al. (2018) well established the links between transport disadvantage, social exclusion and poor health and well-being of older adults. They found that neighborhood built environments in terms of residential density and age-friendly design directly influence their social participation and healthcare access.

In this context, the present study contributed by adding neighborhood proximity and behavior of transport staffs and co-passengers to this model. Here, we have defined neighborhood proximity with contextual factors such as street characteristics, connectivity, and traffic congestion, and not the neighborhood’s design or built environment. In addition, the behavior of the transport personnel also determines the accessibility of transportation for older adults and often influences their travel behavior and thus leads to their social exclusion.

## Data and Methods

The data underpinning the analysis were drawn from qualitative research methods such as visual surveys and in-depth interviews. The data were collected in two stages. First, visual surveys were conducted to observe the challenges of older adults pertaining to access to public transport and mobility in two different neighborhoods in Dhaka. Second, in-depth interviews were conducted in those two neighborhoods. Purposive sampling has been employed to recruit the participants. However, the study is reported as per the COREQ (COnsolidated criteria for REporting Qualitative research) checklist (see supplemental file1) (Torres-Gordillo & Rodríguez-Santero, 2023).

### Participant Recruitment and Profile

In this study, a total of 30 participants aged 60 years and older were recruited to explore the barriers to accessing different modes of transport. The participants were recruited until information saturation, the point at which the information begins to repeat itself. The participants were from two socio-economically different neighborhoods: one is Lalbagh where the residents are mainly engaged in wholesale and retail businesses, government and non-government jobs, and rent out the shops fronting the streets; the second is Rayer Bazaar, which is a low-income household neighborhood. The participants, who were recruited through gatekeepers (such as an NGO called “laughing club” in Old Dhaka and a primary school teacher in Rayer Bazar), were selected to represent a range of living situations and travel requirements (see Table 1). Out of the 30 participants, 14 were from Rayer Bazar (5 women, 9 men) and 16 were from Old Dhaka. Since the researchers involved in this project were only men and due to strong cultural norms and restrictions that limit women’s participation in public life, only male participants were included from Old Dhaka.

**Table 1.** Background of Participants (*N* = 30) With Travel Requirements.

Sl. No	Fictional name	Age	Neighborhood	Occupation	Travel requirements
1	Abdul Hamid	65	Laal Bagh	Small **business owner**	Social interactions and health facilities
2	Abdul Razzak	75	Laal Bagh	Retired bus diver	Social interactions and health facilities
3	Abdur Rahman	72	Laal Bagh	Medical shop owner	Marketing for shop
4	Abdus Salam	76	Laal Bagh	Laundryman	Meeting relatives, health services
5	Abdus Samad	63	Laal Bagh	Small shop keeper	Work, social relations
6	Amjad Hussain	60	Laal Bagh	Medical shop owner	Work, Religious functions
7	Amir Hossain	66	Laal Bagh	Small **business owner**	Marketing, meeting with clients
8	Habibur Rehman	63	Laal Bagh	Ink whole seller	Marketing, meeting with suppliers, social relations
9	Hasan Sheikh	65	Laal Bagh	Works in health department	Work, social relations
10	Aminul Haque	65	Laal Bagh	Prayer leader of a mosque	Health services, social relations
11	Mahabub Alam	62	Laal Bagh	Businessman	Work, meeting with clients
12	Mohammad Ali	62	Laal Bagh	AC mechanic	Work, meeting with clients, dropping niece to college
13	Mohammad Irfan	68	Laal Bagh	Gateman	Work, health services
14	Mujibur Rahman	65	Laal Bagh	Small **business owner**	Working place, social relation
15	Noor Islam	63	Laal Bagh	Tailor shop owner	Marketing for shop, health services
16	Selim Hossain	66	Laal Bagh	Small **business owner**	Meeting with clients, health services
17	Abdul Ahad	66	Rayer Bazar	Construction worker	Work, health services
18	Abdul Gofur	64	Rayer Bazar	Construction worker	Work, health services
19	Anwara	66	Rayer Bazar	Domestic worker	Work, buying grocery
20	Asma	63	Rayer Bazar	Works in a health centre	Work, health services, buying grocery
21	Azizul Islam	64	Rayer Bazar	Electronic shop owner	Social relation, marketing, health services
22	Kanchan Mia	62	Rayer Bazar	Construction worker	Work, buying house stuffs
23	Korful Shekh	65	Rayer Bazar	Works in garment factory	Work, health services
24	Mahmuda	62	Rayer Bazar	Nurse in LabAid Hospital	Work, buying house stuffs
25	Manik Shekh	61	Rayer Bazar	Daily wage laborer	Work
26	Rafiqul Islam	60	Rayer Bazar	Works in transport sector	Social relations, religious activities
27	Rokon Ahmad	60	Rayer Bazar	Construction worker	Health services, work
28	Rowshon Ara	63	Rayer Bazar	Domestic worker	Work, health services
29	Sadek Ali	65	Rayer Bazar	Construction worker	Work, health services
30	Sahara	68	Rayer Bazar	Works in a shop	Work, buying house stuffs

*Note.* All names are pseudonyms.

### Visual Surveys

**Figure 1.**
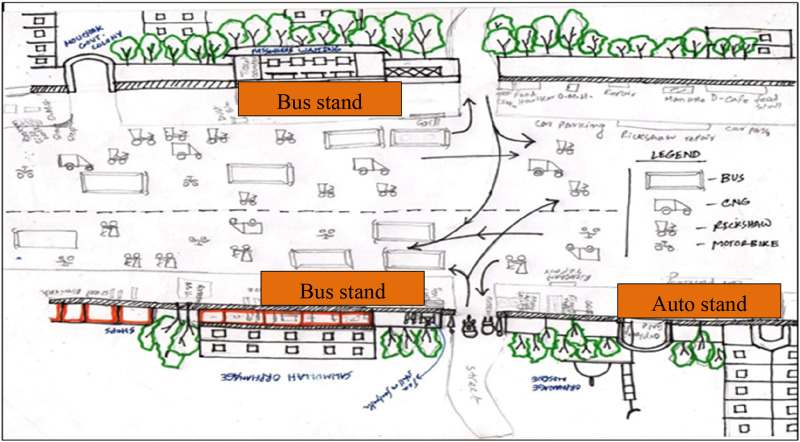
Yatim khana bus stand at Laal Bagh neighborhood. Source: The authors.

**Figure 2.**
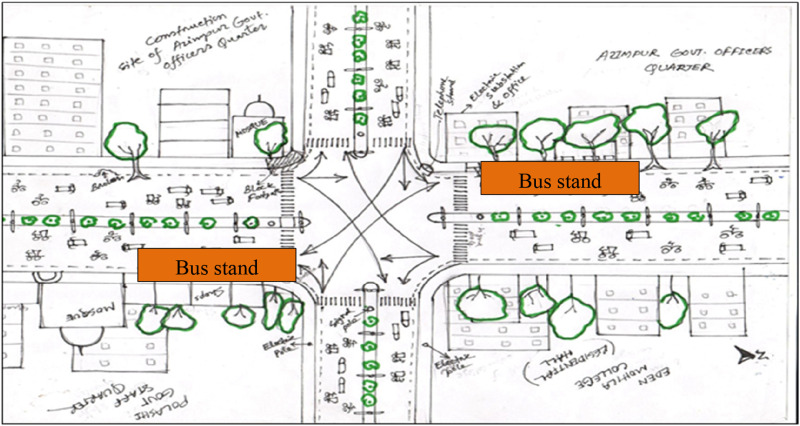
Azimpur bus stand at Lal Bagh neighborhood. Source: The authors.

**Figure 3.**
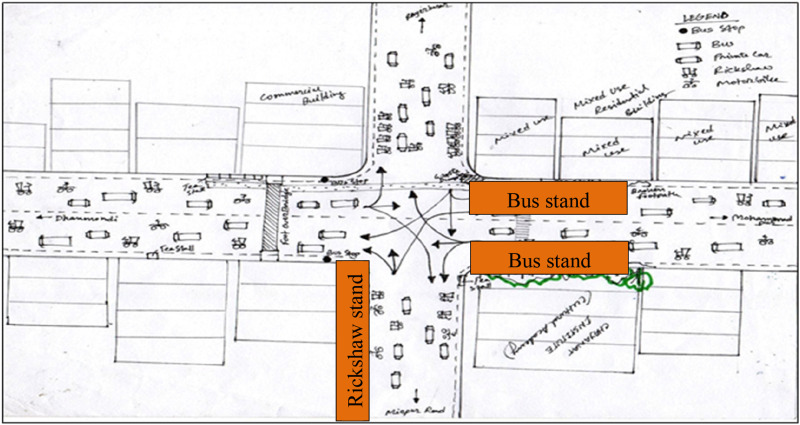
Shankar bus stand at Rayer Bazar neighborhood. Source: The authors.

Visual surveys as a method of qualitative research inquiry add significant insights into the everyday lives of participants (Glaw et al., 2017). Three visual surveys were carried out to map the activities and behaviors of the people and capture the spatial context (Margolis & Pauwels, 2011). The visual surveys were conducted at Yatimkhana and Azimpur bus stands in the Laal Bagh neighborhood (see Figures 1 and 2) and Shankar bus stands in the Rayer Bazar area (see Figure 3). Visual surveys helped to note down various physical barriers of older adults accessing public transport (e.g., boarding and deboarding, struggling to get into crowded vehicles) and activities of people.

### In-Depth Interviews

Thirty semi-structured in-depth interviews were carried out with older adults who used different modes of transportation for their everyday mobility to understand the challenges they face when accessing workplaces, health facilities, and maintaining social interactions (Taylor et al., 2015). A semi-structured in-depth interview guide was used to collect data through personal interviews. Interview guidelines were developed in English, but the interviews were held and translated into Bengali, which was the native language of the participants. Before the main interviews, the interview guides were piloted with two older adults to check for the flow and comprehensibility of the questions, probes, and timing of the interview questions. The interview guide began with opening questions followed by main questions and a few closing questions (see supplemental file 2). The responses revealed ageism in society as a whole and went much beyond concerns about transportation. However, for this study, we only focused on transportation-related issues. The data were collected in Dhaka during January and February 2020, just before the outbreak of the Covid-19 pandemic in Dhaka. Informed consent has been obtained from the older adults for publication of the case report and accompanying images. The interviews were recorded, and the records were fully anonymized while the data were analyzed.

### Data Analysis

The recorded data were transcribed and then translated into the English language for textual analysis. The names of the participants have been pseudonymized in order to protect their privacy. The interviews were analyzed with the help of Atlas.ti 8 in two cycles to accomplish the coding process (Hennink et al., 2020; Saldana, 2011). A thematic analysis approach was adopted to underpin the study. First, deductive codes were developed from the data based on the conceptual framework and objectives of the study. Second, inductive codes were developed directly from the texts (participants’ views) themselves. These codes are the key themes to explain the challenges of accessing public transport in Dhaka. In the second stage, codes were categorized and merged to develop code groups or code families for further analysis (Corbin & Strauss, 2008). We developed code families related to public transport inaccessibility based on our research objectives (see Table 2 for code families and codes with example quotations). Each code was described by comparing different statements and quotes made by the participants.

**Table 2.** List of Code Family and Codes for Older Adults.

Code family	Codes	Frequencies	Example quotations
Availability	Availability of public transport	34	Basically, when I go to the market, I use rickshaw and *CNG* to travel nearby, but when I need to go a long distance, I have to use buses. To avoid traffic jams, sometimes I walk for a long distance; frequently I go to New market by walking (Amjad Hussain, 60, Laal Bagh, medical shop owner)
Behavior	Behaviors towards older adults	22	Their behavior is not good at all. Like, my wife is quite old and she had to go to Ghazipur once to visit her sister. So, she had taken a bus and the place where she was supposed to get down, the driver did not stop bus there. So, she has to get down from the running bus. While getting down she sprained and hurt her leg and now she is bedridden (Habibur Rehman, 63, Laal Bagh, Ink whole seller)
Barriers in public transport	Barriers/experiences in bus	55	The most challenging part about using the bus is while boarding. It is so difficult to board and deboard in a running bus; you almost risk your life (Mohammad Ali, 62, AC mechanic)
	Break journey: Challenges while traveling in buses	26	Buses are available, but sometimes we have to wait. Sometimes direct bus for my destination is not available, so I have to break my journey through changing buses. This is the major problem (Abdur Rahman, 62, medical shop owner)
Social exclusion	Loneliness, social isolation, denial, helplessness	53	I stay back home and do not go outside as much. Sometimes I deliberately evade visiting relatives to avoid traffic. So, I keep up with the relatives through phone calls. Any time if I had to visit my native home back in the village, I prefer to travel during the weekends (Noor Islam, 63, Tailor shop owner)

## Results

The findings reveal that older adults experience several physical (built environment such as footpaths, roads and ramps; traffic congestion; and neighborhood proximity) and social (such as behavior of transport personnel and co-passengers, availability, affordability, low income, accessibility, and frequent denials) barriers in their everyday urban mobility. These barriers have been thematically categorized as “Availability of public transport”; “Behavior towards older adults”; and “Affordability, social isolation, and exclusion.”

### Availability of Public Transport

With winding and intricate streets, these neighborhoods in Dhaka provide multiple options of public transport for the users' everyday mobility. The choices of the modes depend on their requirements for the journey; they take a bus if they need to travel long distances, whereas they use rickshaws for short distances. Some of the older adults mentioned that they also use *Laguna* (a motorized van that carries passengers) and *CNG* (an autorickshaw) to avoid congestion while traveling. In contrast, the majority of the older adults perceived that, though various modes are available, these modes do not operate on time or for desired destinations.

Buses are available, but sometimes I have to stand and wait until I get the bus. If miss any bus, I may have to wait for a long time, sometimes for half an hour for the second bus (Anwara, 66, Domestic workers in Rayer Bazar).

**Figure 4.**
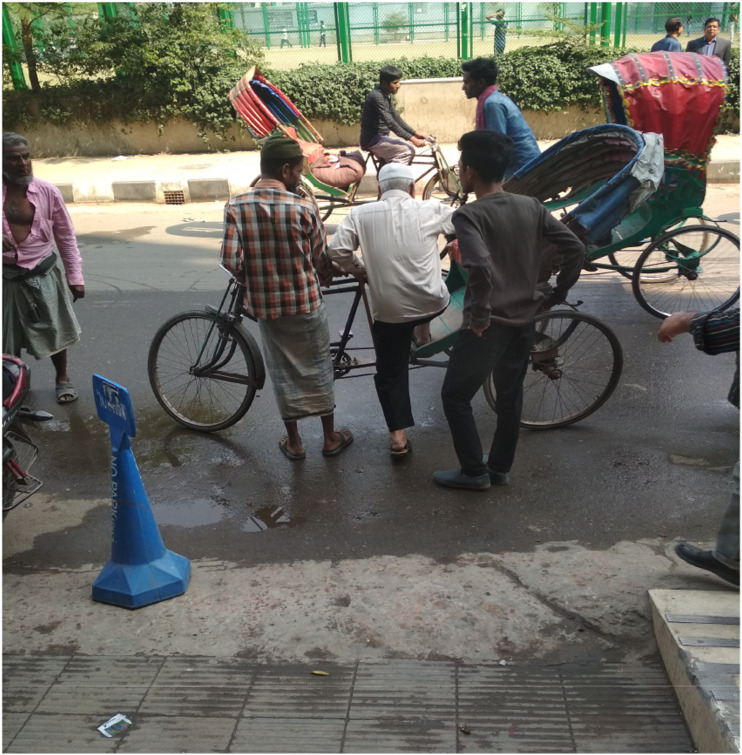
Abdul Hamid getting on a rickshaw after visiting a doctor in Lalbagh neighborhood. Source: The authors.

Moreover, the older adults expressed concerns that the neighborhoods are not directly connected by bus services; they have to take other local modes such as rickshaws and CNG to reach the main bus stands. Both neighborhoods are located far from the main bus stops and reflect horizontal transport inequities. To access buses and other modes of transportation for long-distance travel, they have to take either rickshaws or walk. However, it often becomes difficult to access rickshaws due to their height (see Figure 4). Moreover, walking through congested *chipa rasta* (narrow streets) is risky for older adults, as bikers and autorickshaw pullers drive recklessly and cause frequent accidents. These horizontal transport inequities, thus, limit the everyday mobility of older adults and subsequently exclude them from participating in community activities.

Sometimes the direct bus to my destination is not available, so I have to break my journey by changing buses. This is the major problem (Abdur Rahman, 62, medical shop owner).

Besides, vertical inequities such as old age, gender, low income, and residing in remote neighborhoods have a profound impact on the availability of public transportation. Older adults often require accessible and reliable public transportation that takes into account their specific needs, such as priority seating, assistance, and a safe and convenient boarding process. The lack of these considerations in the transportation system has resulted in their exclusion from public transport as they struggle to navigate the difficulties associated with crowded and risky streets.

It is so dangerous to get on and off a moving bus that you almost risk your life. The buses are always running and one has to board a running bus. Moreover, if a bus has seating for 50 people, 90 people are crammed together; it is so difficult to get down (Mohammad Ali, 62, AC mechanic).

Moreover, older adults from lower-income groups frequently use *Leguna,* particularly in and around Old Dhaka neighborhoods. But traveling in *Leguna* prolongs the journey since people have to wait in a long queue. For instance, Aminul said that he could travel from Gulistan to Azimpur for 15 *taka*, but it would cost 50 *taka* if he traveled by rickshaw.

Using leguna is painful because I have to wait in line for long time. One day I was going Chawkbazar and had to stand after 100 people in the waiting line to get on the leguna (Aminul Haque, 65, Prayer leader of a mosque).

Hence, contextual factors such as neighborhood proximity, which includes lack of last-mile connectivity and subsequent breaking journeys (which prolong journeys), buses not operating on time, and intersectionalities of old age and low income reflect the social dimensions of transport-related social exclusion theory and are embedded in the mobility issues of the older adults in the Global South.

### Behavior Towards Older Adults

The older adults perceived themselves to be excluded while traveling because of the insensitive behavior of the transport personnel and their fellow passengers towards them. The present young generation does not offer seats to older adults in a congested bus. Sometimes the co-passengers physically as well as verbally abuse the older adults, which hurts them the most. Such unwelcome ageist behavior influenced the travel behavior of older adults, often limited their mobility, and thus led to social exclusion. Mahabub Alam, for instance, perceived that he was being excluded from society because people considered him a “*bojha*” (burden).

We, the murubbi (older people), are a bojha (burden) on the people in one way or another; whether it is the people, society, or the city as a whole, they all consider us bojha, wherever we go (Mahabub Alam, 62, Small business owner).

The unsolicited behavior of transport personnel, such as not assisting older adults to board, not ensuring a seat, not stopping the vehicle at the proper location, and denying ridership, is an integral part of the transport-related social exclusion framework and is well reflected in the voices of the older adults.

The CNG auto driver, much like the rickshaw pullers never listens to us. If I ask them to take a particular lane which will the nearest to my destination, they refuse to go. They never listen to the passenger (Selim Hossain, 66, Small business owner, Old Dhaka).

Moreover, gender dynamics further intersected with old age to influence the accessibility of public transportation. Older women in Dhaka face additional challenges due to sexual harassment and social norms that restrict their mobility and interactions with unrelated men. The lack of gender-sensitive transport options and services has exacerbated their exclusion from public transportation.

The male passengers grab those reserved seats. Some of them leave those seats when any female passenger enters the bus. But some ask, “Didn’t we pay money? Don’t you see that there is no space? Why do you ride on such a crowded bus? And they don’t give us the chance to take the reserved seats for female passengers (Mahmuda, 62, Nurse in LabAid Hospital).

### Affordability, Social Isolation, and Exclusion

The majority of the older adults in the Rayer Bazar neighborhood avoided expensive modes of transportation such as the Rickshaw and CNG and traveled in buses despite their frailty so that they could save money to buy food for their families. The low income of the older adults also discourages them from maintaining social relations with their relatives living in other parts of the city. Rokon lives in Rayer Bazar and his cousin lives in Gazipur, but he rarely visits his cousin to avoid “unnecessary” expenditure. If he visits there, it would cost more than 100 *taka* (Bangladeshi currency equal to 1.2USD) on one side because the bus fare is 20 *taka* to Gazipur, and from the Gazipur bus stand, he has to take a CNG or rickshaw that would charge more than 80 *taka* till his cousin’s house. So, he sometimes talks over the phone only. The high cost of transportation services, such as rickshaws or CNGs, often forced them to make trade-offs between essential expenses and mobility. This low-income intersectionality limits their access to public transportation and exacerbates social exclusion.

We are poor people. How can I survive with my family if I don’t go to work? How do I feed my children? I have no alternative. I have to move with these problems (Abdul Gofur, 64, Rayer Bazar, construction worker).

In addition, traffic congestion also affected older adults’ regular social interactions with relatives and friends. To avoid the crowded buses and congestion, older adults avoided social events and canceled visits to relatives. For instance, Nur Islam, who lived at Chowk Bazar Road in the Lalbagh neighborhood, was invited to a wedding ceremony from his cousin’s house in Narayanganj, but he couldn’t go because the program was in the evening and the traffic was too congested at that time, so he dropped the idea to attend that. Non-participation in important events such as marriage and family gatherings push older adults into feelings of loneliness.

Many times, because of traffic jams, we do not go outside, though we want to. Since we have to wait for hours in traffic, we avoid attending relatives’ functions and other social events (Abdul Hamid, 65, a small business owner).

Since older adults do not travel in crowded buses due to their health reasons, they take rickshaws despite high fares. Even though the older adults are from low-income households, they are bound to take rickshaws in such helpless situations. Simultaneously, traffic congestion in urban areas like Dhaka can lead to delays and increased travel times, further straining their limited budgets. As a result, these vertical inequities, such as age, gender, high cost of transportation, and low income not only hinder their access to essential services but also foster social isolation.

## Discussion

Research on transport disadvantage and resulting social exclusion has gained significance in the last two decades in the global North. The majority of research focused on the physical, geographical, and infrastructural dimensions of transport inequity (Duvarci et al., 2015; Luz & Portugal, 2021), whereas social dimensions such as behavior and social intersectionality of age, gender, ableism, and sexuality have rarely been taken into account as part of social exclusion (Lucas, 2012). Like in other Global South cities, the majority of the older people are dependent on public transportation in Dhaka, and very few older people use private cars (Jahangir et al., 2022). Traffic congestion and crowded modes of transport were the major barriers to accessing public transportation for older adults, which discouraged them from participating in workplaces and social interactions. The results of this study intricately implicate transport-related social exclusion by exploring the typical neighborhood characteristics in the Global South: narrow streets, lack of sidewalks, and congested local transport that deprive older adults of accessing work opportunities, health facilities, and social networks and thus lead to isolation and exclusion (Lucas, 2012).

### Lack of Accessibility and Availability of Public Transportation

The physical and infrastructural barriers to accessing public transport induce social isolation and exclusion for older adults as they limit their mobility for fear of fatal accidents. The inadequate infrastructure and transport services, such as not standing properly at the stands, also discourage older adults from traveling to access opportunities (Munshi, 2018). The findings are consistent with the findings of Sohail et al. (2006), who argued that the timely availability of public transport is a major challenge in most developing countries including Pakistan and Sri Lanka. In contrast, Truong and Somenahalli (2015) found that older adults in Australia prefer public transport due to easy access to public transport in neighborhoods. Unusually long waiting times and journeys have an impact on physical and mental health (Mahdavi & Binaei, 2017). Moreover, travel delays also impact working lives; it reduces their efficiency and thus produces a feeling of negativity and isolation (Ma et al., 2018).

The Road Transport Act of 2018 proposed that 30% of seats must be reserved for women, people with disabilities, and older adults in all local buses, but there are no separate seats specifically earmarked for older adults and these regulations are hardly implemented in reality. Moreover, older adults do not have any fare subsidy for bus services in Dhaka. In this context, the policies based on the 4 As framework of Carruthers et al. (2005) would appropriate transport-related social exclusion by entailing accessibility-ease of using transportation for all; availability-route options, schedules, and frequency; affordability-ability to travel without forgoing other necessary activities; and acceptability-impressions of transport influenced by a variety of issues, such as transport staffs’ behavior, the condition of waiting areas, and the vehicle design.

### Low Income and Transport Disadvantage

Economic constraint is one of the major barriers for older adults to access comfortable modes such as the Rickshaw and CNG. Since older adults who were unemployed or worked in the informal sector have to continue working even after 60 years of age for their livelihoods, they are obliged to travel in congested traffic and on crowded public transport, particularly in developing countries (Nadrian et al., 2019). The findings indicated that transportation inequality and social deprivation are closely related, and this is reiterated in the transport-related social exclusion framework, which acknowledges that the mobility of older adults depends not only on the availability and frequency of transport but also on social barriers such as perceptions of affordability, waiting time, fear of crime, traffic facilities, and uncomfortable seating arrangements (Kashfi et al., 2016). A similar study was found in Brazil (Guimarães et al., 2019) and Iran (Panahi et al., 2022), where living locations, travel purpose, and mode design influence older adults’ use of public transport.

### Changing Behavior Towards Older Adults and Exclusion

Undesirable and discouraging social attitudes and behaviors of transport personnel exclude older adults from accessing public transport. Non-cooperation of the transport personnel and co-passengers isolates the older adults while accessing public transport (Tillman et al., 2013). The undesirable behavior of the transport personnel, notably stressed drivers, such as reckless driving to overtake, stops vehicles far from the platform also excludes older adults from accessing public transport as they feel insecure and unsafe traveling in such vehicles (Bezyak et al., 2017). These unwelcome behaviors that affect safety and security while traveling reflect transport-related social exclusion which focuses on how safety and behavior towards older adults determine their mobility. Moreover, walking over congested sidewalks and potential mishaps due to being pushed aside by a young co-pedestrian also produce feelings of disrespect and loneliness. Such social attitudes towards older adults also have an impact on their physical and mental health (Mahdavi & Binaei, 2017).

### Spatial Deprivation and Exclusion

Geographical proximity of neighborhoods with the main junctions, access to essential services, and neighborhood resources have been considered important dimensions of social inclusion because they facilitate older adults' maintenance of social interactions with relatives, friends, and community members (Bissonnette et al., 2012). Previous studies argued that horizontal inequities, such as lower levels of perceived social and physical neighborhood characteristics such as closeness and walkability, produce a feeling of higher levels of loneliness among older adults (Herbolsheimer et al., 2021). The horizontal transport inequity in deprived neighborhoods, in terms of last-mile connectivity and accessing neighborhood resources such as markets, parks for leisure activities, and meeting places for social interactions, in the Global South limits the mobility of older adults and influences the quality of life and well-being (Kearns et al., 2015). This again reflects the transport-related social exclusion model, which argues that the neighborhood can be deprived of availability and access to public transport services even though it does not have a low income. Here, it is mentionworthy that neighborhood deprivation and social exclusion of marginalized groups are not only prevalent in low-income Global South cities but across cultural contexts that do not have first- and last-mile connections (Hine & Mitchell, 2001). The findings of this study also replicate the social exclusion of other marginalized groups, such as women and people with disabilities, living in deprived neighborhoods that do not have safe, accessible, and affordable public transportation.

## Conclusion

This study has significant theoretical and practical implications for the understanding of transportation inequities and social exclusion, particularly among older adults living in densely populated urban areas. By examining the intricate interplay of age, gender, low income, and neighborhood proximity, the study will advance existing theories and inform practical implications in several ways.

### Theoretical Implications

Firstly, our study extends the concept of transport-related social exclusion beyond the traditional understanding of mere physical access to transportation services, such as availability, frequency, and travel time. While these factors remain crucial, we highlight the multifaceted nature of social exclusion within the realm of transportation. Our research underscores that social disadvantage can be driven not only by income but also by factors such as age, affordability, and social attitudes all of which are integral components of transport-related social exclusion. Secondly, our contribution to the theoretical framework emphasizes the role of horizontal transport inequity, particularly in the context of neighborhood deprivation. We argue that neighborhood proximity plays a pivotal role in determining an individual’s mobility, thereby influencing their level of social inclusion or exclusion. Lastly, the inclusion of the behavior of transport personnel, as well as co-passengers, as determinants of accessibility and social inclusion for older adults is a novel addition to the transport disadvantage model. This recognizes that interactions within the transportation system can significantly impact an individual’s experience and ultimately their social inclusion.

### Practical Implications

The practical implications of this study are extensive and can inform policy and practice at various levels, from local to regional and global, to mitigate transportation-related social exclusion among older adults. At the local level, transport authorities can use our findings to develop strategies that address neighborhood proximity as a critical factor in transportation access. Investments in improving street characteristics, connectivity, and managing traffic congestion in neighborhoods can enhance the accessibility of public transportation for older adults. Moreover, the introduction of “age-friendly vehicles” with accessibility features such as low floors, priority seating, and specialized training for bus drivers and conductors can greatly enhance the mobility and dignity of older adults. Furthermore, the provision of ramps and seating areas at bus stops dedicated to older adults can ensure equitable access to public transportation. Additionally, initiatives to raise awareness and promote respectful behavior among transport personnel and co-passengers can foster a more inclusive transport environment.

Looking beyond Dhaka, similarly congested cities in South Asian countries can learn from these practical measures to improve transportation equity for their aging populations. Regionally, our research can serve as a template for other urban areas facing similar challenges. Cities and regions with aging populations can adopt our framework to assess and improve transportation systems, ensuring that they are age-friendly and conducive to social inclusion. On a global scale, our study highlights the universal relevance of the factors contributing to transportation-related social exclusion among older adults. This knowledge can be shared and adapted by cities and regions worldwide, particularly in low- and middle-income countries, where these issues are often more acute due to limited resources and infrastructure.

In short, our research not only advances theoretical understanding but also offers practical solutions to address the transportation-related social exclusion of older adults. By recognizing the significance of neighborhood proximity and the influence of human interactions within the transport system, we provide a roadmap for creating more inclusive and equitable transportation systems globally. These measures can improve the quality of life and social participation of older adults, contributing to a more inclusive and age-friendly society.

### Limitations

However, the study has its limitations. The study focused on neighborhood proximity in terms of street characteristics, connectivity, and traffic congestion, but it did not explore other elements of neighborhood design or the built environment. Future research should consider a more comprehensive examination of neighborhood attributes that influence transportation access and social inclusion.

This study primarily relied on visual survey data and qualitative interviews to gather information from older adults. These methods provided valuable insights, but the cross-sectional and longitudinal studies could have provided more robust evidence of how transportation inequities and social exclusion evolved.

While the study highlighted the role of the behavior of transport personnel and co-passengers, it did not delve deeply into the underlying factors shaping these behaviors. Understanding the drivers of such behavior, including cultural and social norms, could provide more nuanced insights into how to address transportation-related social exclusion.

The number of participants in this study may not fully represent the diversity of older adults in Dhaka. Certain subgroups, such as older women from the Old Dhaka neighborhood, those with severe mobility limitations, or those living in extremely remote areas, have not been adequately represented.

The study’s findings are based on data collected during the winter season, that is, January and February, and transportation systems and urban environments may have different implications in other seasons. It is essential to consider how seasonal changes impact the accessibility of the transportation system in Dhaka.

Participants provided verbal consent before the commencement of data collection and conducting in-depth interviews. The ethics committee formally approved verbal consent. Data records were fully anonymized.

## Supplemental Material

Supplemental Material - “We do not go outside, though We want to”: Unequal Access to Public Transport and Transport-Related Social Exclusion of Older Adults in Dhaka, Bangladesh Supplemental Material for “We do not go outside, though We want to”: Unequal Access to Public Transport and Transport-Related Social Exclusion of Older Adults in Dhaka, Bangladeshin the United States by Selim Jahangir, Ajay Bailey, Musleh Uddin Hasan, and Shanawez Hossain

## Data Availability

Data will be made available on request.
