# Supplementary material for: “We do not go outside, though We want to”: Unequal Access to Public Transport and Transport-Related Social Exclusion of Older Adults in Dhaka, Bangladesh
Source: J Appl Gerontol. 2024 Feb 14;43(8):1165–76. doi: 10.1177/07334648241231156 (PMC11308260; doi:10.1177/07334648241231156)
Supplement: Supplemental Material - “We do not go outside, though We want to”: Unequal Access to Public Transport and Transport-Related Social Exclusion of Older Adults in Dhaka, Bangladesh [file sj-pdf-1-jag-10.1177_07334648241231156.pdf]

**Supplementary File 2: IDI Guide**

## Older adults and barriers to urban mobility in Dhaka
## In-Depth Interview guide

**Introduction:**

*Assalamu Walaikum* (greeting), my name is ******* and I am doing post-doctoral research with *** (masked for review)***.

My research topic is "Older adults and barriers to urban mobility in Dhaka". We would very much appreciate your participation in the interview. We would like to ask some questions regarding your accessibility and mobility for essential services in everyday life. The interview will take between 45-60 minutes. All the information you provide will be used only for research and be kept strictly confidential. Your identity will be anonymised throughout.

Participation in this interview is voluntary and you have the right to stop or deny replying answering questions you find uncomfortable. As part of this interview we would like to ask your permission for recording. We also wish to take photographs to comprehend your experiences and perceptions. So please allow us your consent for conducting and recording the interview.

**Background Information or opening questions:**

1. Can you please tell me something about yourself? Probe: Name, age, education, marital status, employment status, source of income.
2. Which locality of the city do you live in?/since when you are living here? (Probe: For how many years? Why in that particular locality?)
3. What do you feel about Dhaka as a city? How does the city treat you and other older adults? (Probe: too congested, traffic, pollution, (un)friendly, risky, inclusive, indifferent, alienated).

**<u>Household and neighbourhood</u>**

4. How many members do you have in your family? Probe (type of residence, ownership of house, number of vehicles in the house).
5. Can you tell me about your social life (probe: extended family members, fellow older adults, friends, neighbours, leisure activities).

**Key Questions or Main Questions:**

6. What does your normal day look like? (Means what is your daily routine)?

(Probes: Bathing, clothing, eating, reading newspaper, outing for work place, domestic need, health care)

7. What are your travel requirements? Probe: (How often you go out? How far, Why do you go out?)
8. What modes of transport do you use and for what purposes?

9. What transport you used in the past and why are you not using it now?
10. Do you need someone to accompany during your travel? If yes why do you need to accompany you?

<u>Questions on physical challenges</u>

11. What are the physical difficulties/barriers you face while walking on the streets?

Probe: Footpaths – lack of sidewalk, slope, surface condition, length, steps, width and traffic; foot over bridge, subway, ramps, curb, lighting, tactile paving, zebra crossings, traffic signage

12. What measures would you suggest to improve your walking/moving experience?

**Barriers in public transport:**

13. What are the difficulties/barriers you face while travelling in public transport?

(Probe: while accessing bus, train, rickshaws, metro)

***Accessibility Barriers:***

14. What public transport can you access? and how many of them you use?

(Probe: from home to destination, from destination to back home)

15. Could you please share your experience of physical challenges while accessing public transport?
17. What are the main difficulties in accessing such transport for you?

(Probes: proper stands, within easy walking distance, waiting centres, vehicle design).

18. What time do you prefer to travel and why? (probe: day time, afternoon, evening, night).

*<u>Access to workplace.</u>*

19. How do you access your workspace? (Probe: mode, the time required to travel)

20. What are the modes of transport you use to reach your work place? (Prove: Rickshaw, auto, bus, metro)

22. What are the changes you experienced over the years regarding travel to access work place? (Probe: travel time, traffic jam, increasing vehicles and crowd).

23. How your work life has affected due to difficult access to work place?

(Probe: reduced work efficiency, re-location of residences, reduced social activities).

*<u>Access to health care.</u>*

24. How is your health condition now? (Probe - any chronic diseases, any kind of disability, multiple morbidity).

25. What barriers you face accessing healthcare through existing means of transport?

(Probe: distance, no direct connectivity, unavailability of public transport)

Who accompanies you to the hospital and why?

26. What are the impacts of transport inaccessibility on your health?

(Probe: psychological stress, discomfort, degrading health condition)

**Affordability Barriers:**

27. How far the mode of transport you use is affordable or expensive?

(Probe: clear fare structure, chart in vehicles/stands)

28. What kind of concession do you get in transportation?

(Probe: how much, do you need cards, where do you get the concession cards, how far is that card centre, how much does it cost, what is the validity of such concession cards?)

**Availability Barriers:**

29. How frequent are the services? (Probes: waiting time, whether on time, frequency)
30. Can you access reserved seats? (Probes: Do the vehicles have reserved seats? Do people get up when you get in).
31. How do you reach your destination or back home?
(Probes: Home to terminals, bus stand to destination point and back).

**Acceptability Barriers**:

32. How do you feel traveling by these modes?
(Probes: seat condition, road condition, jerking, safe, risky due to rash driving)
33. How do you find the behaviours of the transport personnel?
Probe: skill and knowledge, way of speaking and body language)
34. How reliable are these public transports for you?
(Probe: responsible, timing, sincere to passengers)
35. What time of the day do you prefer to use the public transport? Why?
(Probe: visibility, weather condition, peak hours).

**Barriers in private transport:**

36. Why do you use private transport for your everyday mobility? (Probes: affordable, comfort, status, time saving).
37. What are the challenges you face using private transport?
Probes: condition of roads, use of flyovers, being honked at for slow driving, problem of vision, traffic congestion, traffic signals, parking issues, distance between parking and destination, due to other transport users.
38. How safe do you feel driving around? Why? What time do you prefer to travel and why? (probe: day time, afternoon, evening, night).

*Questions on social barriers to access the transport facilities*

39. What are the other challenges do you face in using transport facilities?
(Probe: Family restrictions, Health does not permit to travel, Economic factors- poor household income, high fare, Traffic congestion, Insensitivity of transport personnel, Fear of fatal accidents, Hesitation due to low educational attainment)
40. What are the impacts of these barriers on your social life?
(Probe: On health care facilities including mental health, Social network and participation, Social exclusion and loneliness).
41. What are the impacts of such barriers in your economic life?
(Probe: Confined within limited opportunities, reduced the productivity, delay in reaching workplace, low wages, rendering perpetual poverty).

**<u>Questions on motility</u>**

Questions on **Accessibility** have already been asked.

**Transport competence:**

42. How efficient are the transport you use? (Probe: able to reach on time, adequate knowledge of transport personnel, are they technically skilled?)
43. How do you find the behaviours of the transport personnel? (Probe: flexible, cooperate, express concern).
44. What about the quality of transport management? (Probe: innovations, sensitizing employees, seek suggestions for improvement).

**Appropriation of mobility (Measures to improve mobility)**

45. How do you cope with the challenges you face in everyday mobility? (Probe: Challenges to work, health care facilities and social interactions).
46. Who supports you to cope with these challenges? (Probe: Family support, Co passenger's assistance).
47. How do your household members feel/acknowledge the barriers you have mentioned above? (Probe: Encourage, suggest to stay back home, role do they play to reduce or remove the barriers, how effective is their role).
48. How do your society/neighbourhood members or persons you meet/encounter everyday feel/acknowledge the barriers you have mentioned above? (Probe; what role they play, how effective is their role)
49. What changes do you want to see to minimize the transport difficulties?
Probe: urban design-roads, footpaths, pavements; Bus stops and terminals; Access to different modes of transportation; promoting cycling; Reducing traffic congestion; Parking facilities; Underpasses and over bridges; Renewing driving license
50. What changes do you want to improve the accessibility of work place, health care centres and social interactions?
(Probe: change in mode of transport, government policies, community participation).

**Closing Questions:**

51. What do you think is the main reason behind this unequal mobility? (Probe: Role of transport authorities and government, attitudes of transport personnel).
52. What is your overall impression about the city's future? (Probe: inclusive, sensitive).
53. May I come once more for further clarification in future?

If yes, can we have your phone and email address?

Then can we close interview here?

Okay. Thank you very much for sharing your ideas and perceptions on this.

**Supplementary File 1**

**COREQ (COnsolidated criteria for REporting Qualitative research) Checklist**

A checklist of items that should be included in reports of qualitative research

| **Topic** | **Item No.** | **Guide Questions/ Description** | **Author Responses** |
|---|---|---|---|
| **Domain 1: Research team and reflexivity** | | | |
| *Personal Characteristics* | | | |
| Interviewer/facilitator | 1 | Which author/s conducted the interview or focus group? | First author [Anonymized] |
| Credentials | 2 | What were the researcher's credentials? E.g. PhD, MD | PhD |
| Occupation | 3 | What was their occupation at the time of the study? | Postdoctoral Research Fellow |
| Gender | 4 | Was the researcher male or female? | Male |
| Experience and training | 5 | What experience or training did the researcher have? | Well trained and experienced in conducting in-depth interviews and visual survey.<br>He is the coordinator of Certificate Course in Qualitative Research Methods at [Anonymized], Manipal, India |
| *Relationship with participants* | | | |
| Relationship established | 6 | Was a relationship established prior to study commencement? | Yes, prior rapport was developed before the interviews. |
| Participant knowledge of the interviewer | 7 | What did the participants know about the researcher? e.g. personal goals, reasons for doing the research | Personal goals and reasons for doing the research. |
| Interviewer characteristics | 8 | What characteristics were reported about the inter-viewer/facilitator? e.g. Bias, assumptions, reasons and interests in the research topic | Reasons and interests in the research topic. |
| **Domain 2: Study design** | | | |
| *Theoretical framework* | | | |
| Methodological orientation and Theory | 9 | What methodological orientation was stated to underpin the study? e.g. grounded theory, discourse analysis, ethnography, phenomenology, content analysis | Thematic analysis (see data analysis section) |
| Sampling | 10 | How were participants selected? e.g. purposive, | Purposive sampling (see Data and Methods section) |

| | | | |
|---|---|---|---|
| | | convenience, consecutive, snowball | |
| Method of approach | 11 | How were participants approached? e.g. face-to-face, telephone, mail, email | Face-to-face (see In-depth Interviews section) |
| Sample size | 12 | How many participants were in the study? | 30 participants |
| Non-participation | 13 | How many people refused to participate or dropped out? Reasons? | 2 participants (apart from above mentioned 30) were dropped because of their inability to express views due to frailties. |
| *Setting* | | | |
| Setting of data collection | 14 | Where was the data collected? e.g. home, clinic, workplace | Home and workplace |
| Presence of non-participants | 15 | Was anyone else present besides the participants and researchers? | No |
| Description of sample | 16 | What are the important characteristics of the sample? e.g. demographic data, date | Demographic data |
| *Data collection* | | | |
| Interview guide | 17 | Were questions, prompts, guides provided by the authors? Was it pilot tested? | Yes. An In-depth interview (IDI) guide including questions and probes was provided by the author.<br>The IDI guide was pilot tested with 2 participants. |
| Repeat interviews | 18 | Were repeat interviews carried out? If yes, how many? | 2 repeat interviews were carried out. |
| Audio/visual recording | 19 | Did the research use audio or visual recording to collect the data? | The research used audio recorder to collect the data. |
| Field notes | 20 | Were field notes made during and/or after the interview or focus group? | Yes. The field notes were made during and after interviews. |
| Duration | 21 | What was the duration of the interviews or focus group? | 45 minutes to 1 hour |
| Data saturation | 22 | Was data saturation discussed? | Yes (see Participant recruitment and profile section). |
| Transcripts returned | 23 | Were transcripts returned to participants for comment and/or corrections? | Yes |
| **Domain 3: analysis and findings** | | | |
| *Data analysis* | | | |
| Number of data coders | 24 | How many data coders coded the data? | 1 |
| Description of the coding tree | 25 | Did authors provide a description of the coding tree? | Yes |

| | | | |
|---|---|---|---|
| Derivation of themes | 26 | Were themes identified in advance or derived from the data? | Both deductive and inductive codes were developed from data. |
| Software | 27 | What software, if applicable, was used to manage the data? | Atlas.ti8 |
| Participant checking | 28 | Did participants provide feedback on the findings? | yes |
| *Reporting* | | | |
| Quotations presented | 29 | Were participant quotations presented to illustrate the themes/findings? Was each quotation identified? e.g. participant number | Yes |
| Data and findings consistent | 30 | Was there consistency between the data presented and the findings? | Yes |
| Clarity of major themes | 31 | Were major themes clearly presented in the findings? | Yes |
| Clarity of minor themes | 32 | Is there a description of diverse cases or discussion of minor themes? | Yes |

Developed from: Tong A, Sainsbury P, Craig J. Consolidated criteria for reporting qualitative research (COREQ): a 32-item checklist for interviews and focus groups. *International Journal for Quality in Health Care*. 2007. Volume 19, Number 6: pp. 349 – 357

**Additional Guidelines for Completing the COREQ Checklist for *Journal of Applied Gerontology*:**

- This checklist will be published online as supplementary material and we require it to be in the form of a publishable table. Please make sure that material does not bleed outside of cells, etc.
- This checklist is designed to direct readers to relevant material in the manuscript. Where applicable, please direct readers to various sections of the manuscript, such as a Methods section, Conceptual Framework, table or figure. Pages may shift during the publication process so please avoid directing readers to specific page numbers.
- This checklist also is designed to supplement information that may not be reported in the text and/or provide additional details related to information that is reported in the text.

**Once you have completed this checklist, please save a copy and upload an <u>anonymized version</u> of it as part of your *Journal of Applied Gerontology* submission. DO NOT include this checklist as part of the main manuscript document. It must be uploaded as a separate supplemental file. If the paper is accepted, a non-anonymized version should be provided with the final submission of the main manuscript.**
